# Pulmonary Lymphangitic Carcinomatosis: An Atypical Presentation Leading to Discovery of Multi-Organ Metastasis With Unknown Occult Primary Malignancy

**DOI:** 10.7759/cureus.27705

**Published:** 2022-08-05

**Authors:** Mustafa Al-Bayati, Ola Al-Jobory, Falah Abu-hassan, Basheer U Mohammed, Sinan Yaqoob, Omar Bazzaz

**Affiliations:** 1 Internal Medicine, Texas Tech University Health Sciences Center, Amarillo, USA; 2 Internal Medicine, Thomas E. Creek VA Medical Center, Amarillo, USA

**Keywords:** pulmonary disease, oncology, liver masses, pulmonary metastasis, lymphangitic carcinomatosis, pulmonary lymphangitic carcinomatosis, plc

## Abstract

Pulmonary lymphangitic carcinomatosis is the spread of malignant cells to the lymphatic system in the lungs, which results in inflammation and lymphatic dilation. It is often found to coexist in patients with prior history of malignancy. The clinical presentation is usually related to respiratory symptoms, but atypical presentation can occur. Chest x-ray imaging can be non-revealing at initial stages, and abnormalities may only be appreciated when the disease gets to more advanced stages. Computed tomography imaging can reveal radiological abnormalities that were found to be associated with pulmonary lymphangitic carcinomatosis. More advanced imaging modalities and pathological tissue confirmation may be required for the diagnosis. However, once the diagnosis is made, prognosis remains poor and treatment efforts are geared towards the underlying malignancy. Here, we report on a rare case of pulmonary lymphangitic carcinomatosis in an adult male with no prior history of cancer, for whom his hospitalization led to the discovery of malignancy involving multiple organs.

## Introduction

Pulmonary lymphangitic carcinomatosis (PLC) is defined by the presence of malignant cells in the lymphatic system within the pulmonary circulation. It was first documented by Dr. Gabriel Andral in 1829 in a patient with uterine malignancy [1]. It is a rare disease as evidenced by only having 65 published papers during the period of 2000-2018 [2]. It presents most commonly with shortness of breath among other respiratory complaints. It was observed that most patients have a prior history of malignancy, however in rare instances, PLC can be the initial presentation of an occult malignancy. 

## Case presentation

A 53-year-old male with past medical history of chronic alcohol abuse, benign prostatic hyperplasia, and hypothyroidism, was admitted to our hospital for complaints of abdominal pain and constipation. Patient reported having abdominal pain for a few weeks that was present continuously, in addition to constipation and trouble urinating for the same duration. He also mentioned having mild dry cough and nonspecific chest discomfort. Further history was significant for anorexia and weight loss of about 25 pounds within the last three months prior to his presentation. He is a smoker for more than 30 years and drinks alcohol heavily on a regular basis. Family history was significant for an unknown cancer in his mother. On physical examination, vital signs were within normal range and maintaining normal oxygen saturation on room air. He had normal heart sounds and lungs were clear to auscultation bilaterally and the abdomen was soft with marked hepatomegaly with a liver span of 14 cm. Lab workup showed mild anemia of 13.7, moderate hyponatremia of 126, mild hypercalcemia of 11.7, albumin 3.6, creatinine of 0.91, total bilirubin of 1.1, aspartate aminotransferase (AST) of 97 and alanine aminotransferase (ALT) of 51. Other relevant lab studies during the hospital stay include alkaline phosphatase of 366 with gamma-glutamyl transferase (GGT) of 585, parathyroid hormone (PTH) 11.4, lactate dehydrogenase (LDH) 244, carcinoembryonic antigen (CEA) 36.8, alpha fetal protein 40.1, cancer antigen 19-9 (CA 19-9) 7, free prostate-specific antigen (PSA) 0.2, total PSA 1.2, thyroid-stimulating hormone (TSH) 11.5, T4 0.98, negative stool occult blood, negative tuberculosis (TB) QuantiFERON. Hepatitis panel was noted to be negative.

**Figure 1:**
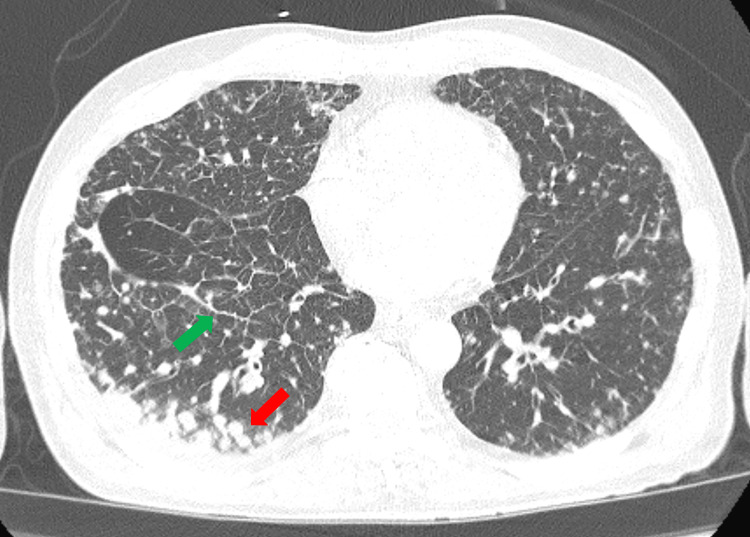
Contrast enhanced computed tomography scan of the chest showing multiple pulmonary nodules (red arrow) within lower lung fields, in addition to intralobular septal thickening (green arrow) in a nodular fashion.

Because of his abdominal complaints, CT abdomen without contrast was obtained which showed innumerable pulmonary nodules within lower lung fields with the largest being 1.6 x 1.9 cm in addition to intralobular septal thickening in a nodular fashion, in addition to pericardial lymphadenopathy, hepatomegaly measuring 24.4 cm with mild hepatic parenchymal heterogeneous attenuation without definite focal lesion identified, in addition to prominent retroperitoneal lymphadenopathy. To further investigate lung abnormalities, CT thorax and abdomen and pelvis with and without contrast (Figure 1) was done and showed similar findings to previous CT and reported many of the pulmonary nodules were in pleural/subpleural distribution and with areas of interlobular septal thickening and nodularity, along with mediastinal and bilateral hilar lymphadenopathy. Liver showed a nodular contour with heterogeneous enhancement on arterial phase, and questionable subcentimeter hypodensities within the liver, in addition to a prominent upper abdominal and retroperitoneal lymphadenopathy, small focal lucency adjacent to the right pedicle of the L3 vertebral body. Because the CT scan was showing a lytic lesion adjacent to L3 vertebra and there was hypercalcemia during his hospitalization with a corrected calcium of 12.5, it was thought that the patient possibly had vertebral metastasis. Whole-body bone scan was ordered to further evaluate, and the results were unremarkable without any significant findings.

**Figure 2:**
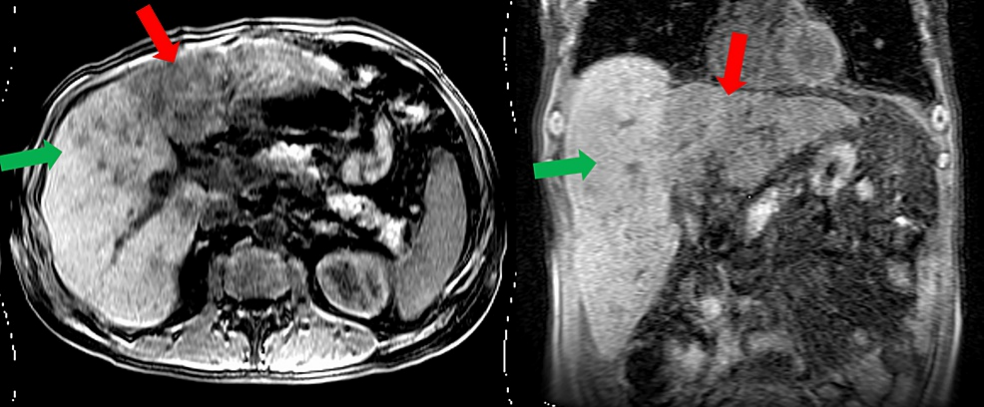
Magnetic resonance imaging of the liver in axial (left) and coronal (right) sections showed liver cirrhosis with widespread signal abnormality throughout the liver. Infiltrative hepatocellular carcinoma of the left and caudate lobes (red arrow) with intrahepatic metastases to the right lobe (green arrow) not excluded per radiology report.

Abdominal MRI (Figure 2) was done without contrast (due to patient fears after having an allergic reaction to CT contrast) which showed liver cirrhosis with widespread signal abnormality throughout the liver. Infiltrative hepatocellular carcinoma of the left and caudate lobes with intrahepatic metastases to the right lobe not excluded per radiology report. CT head was negative for intracranial mass. Echocardiogram was normal without pulmonary hypertension.

To further evaluate the primary malignancy source, fluorodeoxyglucose (FDG)-positron emission tomography (PET)/CT scan was set up to be done, however, the patient was having anxiety attacks and fear from his newly found medical condition and opted not to undergo the scan. CT-guided biopsy of a right lung nodule was done and reported as the following; "Immunohistochemical studies do not definitively pinpoint the origin of the tumor. The presence of CDX2, MUC5AC, IMP3, and TTF-1 (weak-patchy) may be compatible with an enteric-type lung primary adenocarcinoma. The differential diagnosis would also include metastasis from pancreatobiliary or gastrointestinal tumors or adenocarcinoma of other sites with enteric-type differentiation".

Up to this point, oncology were following and the patient was given a survival time of 30 days in light of all the findings. The patient elected to go back to his family in his home state and continue follow-up with oncology there. Within a week of discharge from our hospital and a few days prior to his appointment at his home state, patient was hospitalized again for spontaneous bacterial peritonitis and abdominal ultrasound was done showing right lobe liver mass of 4.5 x 2.9 x 3 cm. Additional right lobe masses measuring 1.6 cm, 1.9 cm, and 1.8 cm, and questionable isoechoic caudate lobe mass 5.7 x 5.8 x 3.5 cm. This imaging pattern was worrisome for multifocal hepatocellular carcinoma (HCC) versus metastatic disease per the radiology report. Patient also had significant ascites and paracentesis was done draining 4L with analysis of ascitic fluid returning exudative, negative for malignant cells and showing reactive mesothelial cells. Patient was treated with antibiotics, and he was unsure about his decision to pursue chemotherapy. After a few days, he decided on and was discharged to home hospice.

## Discussion

Pulmonary lymphangitic carcinomatosis is defined as the spread of malignant cells to the lymphatic system within the pulmonary circulation, which subsequently leads to inflammation. The primary tumors that are usually associated with PLC include breast, lung, and gastrointestinal tract, among others. It can be found in up to 8% of metastasis to the lung [3]. The pathophysiology is poorly understood, but access to the lymphatic system is thought to be by direct invasion, lymphatic circulation, or hematogenous spread. Once the pulmonary system is reached, it has been proposed that this subsequently leads to obstruction and distention of lymphatic channels, and invasion of the tumor cells into the adjacent interstitium [4,5]. As a result, the intralobular and interlobular septa become thickened secondary to tissue edema [1].

PLC often presents in the fifth decade in adults, equally in males and females. The symptoms experienced among patients according to a meta-analysis were in the following frequency: dyspnea 59%, dry cough 34%, weight loss 17% [2]. The typical clinical scenario is for PLC to be discovered in a patient with an already established history of malignancy. However, in rare cases, it can be the first presentation leading to the discovery of malignancy. Chest x-ray imaging can be unremarkable in up to 50% of patients. With time, when the disease becomes more advanced, reticulonodular changes can be seen especially in the lower lobes [1]. Chest CT scan done in earlier stages of the disease shows "interstitial lesions, linear and reticular shadows and interlobar fissure thickening" [3]. When more advanced disease stages are reached, imaging can show "irregular thickening of the tracheal vascular bundles and interlobular septa, as well as multiple beaded, small nodules of varying sizes (usually smaller than 3 mm in diameter) distributed along the interlobular septa and pleura" [3]. Out of all the CT findings observed in PLC patients, peribronchovascular thickening is considered to be the most significant. As it was found to have an odds ratio (OR) 10.95 (3.33-36.0) P <0.001 for association with PLC, with a sensitivity of 0.69 and a specificity of 0.83 [6]. FDG PET/CT imaging can further confirm the diagnosis by showing increased tracer uptake in the areas previously identified on CT that had peribronchovascular thickening [6]. FDG PET/CT was found to have a specificity of 100% and a sensitivity of 86% for identification of PLC [7]. The utilization of high-resolution computed tomography (HRCT) was compared to FDG PET/CT for the diagnosis of PLC in lung cancer patients, and it was found that both modalities performed similarly for the diagnosis of PLC [6]. The diagnosis of PLC is often delayed by months from the onset of symptoms, since the usual respiratory symptoms are common and do not warrant extensive work-up on initial presentation, and also the patient's age is less than that of a lung cancer patient. In addition, when imaging such as chest x-ray or CT are carried out, findings may be misinterpreted for interstitial lung disease (ILD) diagnosis including sarcoidosis, therefore further diagnostic and therapeutic intervention will be aimed at that incorrect diagnosis until the appropriate diagnosis is reached [8].

Histopathologic confirmation is sought after to finalize the diagnosis of PLC. This can be obtained through a less invasive transbronchial approach, or a more invasive open lung biopsy approach. The more invasive biopsy methods come with procedural risks that need to be considered when such a decision is made [1]. Therefore, on many occasions, the diagnosis is made by combining the clinical presentation along with imaging findings. Currently, there are no therapeutic interventions aimed specifically for PLC. Rather, management is largely supportive and directed at the underlying malignancy with chemo-radiation and surgical resection as feasible [4]. There are isolated reports of using "tyrosine kinase inhibitors (e.g., apatinib), and certain monoclonal antibodies (e.g., bevacizumab, cetuximab)" [1]. Once the diagnosis of PLC is made, prognosis remains poor. The majority of patients are given a survival time of less than six months [3]. However, trends of survival time have shown some improvement during recent years.

In our case, although histopathological evaluation did not pinpoint the exact primary malignancy, a case for diagnosing PLC can be made based on the clinical presentation and radiological findings. As mentioned earlier, the diagnostic role of imaging modalities such as HRCT and FDG PET/CT was studied in PLC patients, and it was found that both modalities performed similarly and reliably for the diagnosis of PLC. Therefore, this served as the basis of diagnosis in this patient. 

## Conclusions

In summary, the low incidence, relatively younger patient population, atypical clinical presentation, and nonspecific radiological findings are the main reasons for delay and misdiagnosis of pulmonary lymphangitic carcinomatosis. Patients commonly present with respiratory complaints including shortness of breath and cough. Such complaints usually warrant a simple imaging modality such as x-ray which may not be revealing. And so, the diagnosis may be delayed until the disease is more advanced and better imaging modalities are utilized. PLC occurs as a result of the spread of primary malignancy most commonly lung, breast, and gastrointestinal tract. Definite diagnosis requires tissue pathology which may not always be feasible depending on the patient's overall condition. However, on many occasions, it is a clinical diagnosis combining patient presentation and radiological findings in reaching the diagnosis. Treatment options are mainly supportive and aimed at addressing the primary malignancy. Once the diagnosis is made, prognosis remains poor and patients are often given a survival time estimated at a few months. 
